# Influence of intravenous 10% amino acids infusion on serum albumin concentration in hypoalbuminemic dogs

**DOI:** 10.3389/fvets.2023.1198534

**Published:** 2023-06-05

**Authors:** Sabrina Schneider, Katrin Hartmann, René Dörfelt

**Affiliations:** Clinic of Small Animal Medicine, Centre for Clinical Veterinary Medicine, LMU Munich, Munich, Germany

**Keywords:** albumin synthesis, canine, survival, parenteral nutrition, hypoproteinemia

## Abstract

**Objective:**

To evaluate the effect of parenteral amino acid application in hospitalized hypoalbuminemic dogs.

**Materials and methods:**

Medical records of client-owned hypoalbuminemic dogs (albumin ≤ 25 g/L) were analyzed. Dogs receiving amino acids for only 1–2 days, receiving transfusions or surgery, or <6 months of age were excluded. Dogs were grouped as those receiving intravenous amino acids (AA, 80 dogs) over 3 days and longer, and those without additional amino acid treatment (CON, 78 dogs). Duration of hospitalization, albumin, and total protein concentrations were compared between groups by Mann–Whitney U test. Course of albumin and total protein concentration was evaluated by Friedman test and Dunn’s multiple comparison test. Significance was set to *p* ≤ 0.05.

**Results:**

Dogs in group AA received 10% amino acid solution intravenously over median 4 days (3–11 days). No significant differences regarding survival and adverse effects were observed between groups. Dogs of group AA had significantly longer duration of hospitalization (median 8 days; 3–33 days) compared to group CON dogs (median 6 days, 3–24 days; *p* < 0.001). Initial albumin concentration was lower in group AA compared to CON (*p* < 0.001). This difference was no longer present on day 2 (*p* = 0.134).

**Conclusions and clinical relevance:**

Intravenous application of 10% amino acid solution in hypoalbuminemic dogs can improve albumin concentration after 2 days, but does not influence outcome.

## 1. Introduction

Albumin is synthetized in the liver from amino acids and fulfills multiple physiologic tasks, such as maintaining colloid osmotic pressure and being a transporter of enzymes, hormones, and pharmacological substance. It is distributed in the plasma (40%) and the interstitium (60%) (1–4). Albumin is also included in the endothelial glycocalyx, preventing fluid shift from the capillaries to the interstitium. Furthermore, it decreases the cytotoxic effect of reactive oxygen species (5, 6).

Hypoalbuminemia is a common disorder in critically ill patients (1). A serum albumin concentration below 20 g/L is associated with decreased survival in a variety of diseases, including protein-losing enteropathy and nephropathy, liver failure, sepsis, heart failure, and hemorrhage (1, 7–10). Protein-losing enteropathy subsumes various gastrointestinal diseases in which protein loss due to damage to the mucosa or epithelium occurs, for example due to inflammatory processes, due to lymphatic obstruction or altered vascular permeability (11, 12). In systemic inflammation and sepsis, trauma or inflammation, the synthesis of the negative acute-phase proteins, including albumin, is reduced (2, 13).

Hypoalbuminemia contributes to life-threatening complications, such as systemic organ failure, pulmonary edema, reduced wound healing, and hypercoagulability (1).

As hypoalbuminemia is often a result of an underlying disease, the treatment should primarily address the underlying process. Measures to increase albumin concentration as well as replacement of albumin function have to be initiated in patients with severe hypoalbuminemia (1). Natural colloids, such as plasma and human or canine albumin, can be used to increase albumin concentration (14). About 45–50 mL/kg plasma are required to increase serum albumin concentration by 10 g/L (15, 16). Human albumin is more effective than plasma to elevate albumin concentration, colloid osmotic pressure, total protein as well as systemic blood pressure (17–20). Mild to severe adverse effects, such as immediate anaphylactic reactions, delayed type III hypersensitivity reactions and development of anti-human albumin antibodies, are reported in dogs after application of human albumin (20–22). Canine albumin also has been shown to increase albumin levels in dogs with potentially fewer adverse effects compared to human albumin (23). However, it is more expensive than human albumin and not available in most countries.

In mechanically ventilated humans, an adequate protein intake adapted on calculations using repeated indirect calorimetry measurement, was found to reduce mortality compared to patietns with daily equal protein intake (24). The American Society for Parenteral and Enteral Nutrition recommends an adequate protein supply for adult critically ill patients (1.2–2.0 g/kg body weight per day) (25). In a worldwide survey on critically ill patients, the median protein intake was only 1.3 g/kg/d (1.0–1.5 g/kg/d), which is at the lower end of the required protein quantity (26). To support albumin synthesis in hypoalbuminemic dogs, enteral and parenteral nutrition is often performed (1). In rats, enteral nutrition improved the albumin transcription rate (27). Enteral nutrition is preferred over parenteral nutrition, as it mimics the physiological situation and supports the function of the gastrointestinal tract (28). As it is often difficult to apply the full amount of proteins and amino acids to critically ill patients, parenteral administration of amino acids can be used to support protein balance (29). In human medicine, parenteral administration of amino acids in addition to enteral nutrition increases synthesis rate and improves protein balance in a catabolic metabolic phase. However, an increase in absolute serum albumin concentration has not been observed in those critically ill humans receiving parenteral amino acid supplementation. Redistribution of albumin molecules between the intra- and extravascular space is considered responsible for the lack of albumin increase (29–32). On the other hand, the administration of amino acids in combination with lipids to premature infants and piglets showed a significant increase in albumin synthesis rate at high levels of amino acid support, with a partially positive effect on serum albumin concentration (33–37). In small animal medicine, the effect of parenterally administered amino acids on albumin synthesis has not been intensively studied.

Aim of this study was to evaluate the effect of parenteral amino acid application in hospitalized dogs suffering from various diseases causing hypoalbuminemia. This study specifically aimed to evaluate the effect of amino acid application on survival, duration of hospitalization as well as on serum albumin and total protein concentration and time to albumin concentration increase. Correlation of the amino acid dose with increase of serum albumin concentration and adverse effects of amino acid application were investigated in comparison to dogs without amino acid supplementation.

## 2. Materials and methods

### 2.1. Study design and animals

**Figure 1:**
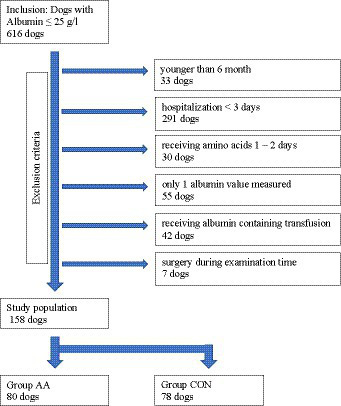
Flow chart on hypoalbuminemic dogs included and excluded in the study.

Medical records of dogs presented to a university clinic from 2013 to 2019 were reviewed for albumin concentrations ≤25 g/L. Dogs were excluded if they were younger than 6 months, hospitalized for less than 3 days. if albumin was only measured once during hospitalization, if they received plasma, whole blood or human albumin transfusion or if they underwent a surgical procedure (other than placement of feeding tubes) during the examination period. Dogs receiving amino acid infusion for only 1–2 days were also excluded (Figure 1).

### 2.2. Study groups

Of the 158 included dogs, 80 received 10% amino acids as a constant rate infusion (Aminoplasmal^®^ 10%, B. Braun, Melsungen, Germany; group AA) for ≥3 days, and 78 dogs were treated without intravenous amino acids (group CON). Signalment, diagnosis, initial and repeated albumin values, total protein values, systemic inflammatory response syndrome (SIRS) parameters, amount of amino acid application, enteral nutrition by feeding tubes, duration of hospitalization, and survival to discharge were recorded in all dogs. SIRS was diagnosed when ≥2 of the 5 SIRS criteria (heart rate, respiratory rate, rectal temperature, amount of white blood cells and percentage of band white blood cells) were fulfilled as described elsewere (38, 39).

### 2.3. Statistical analysis

Statistical analysis was performed with a commercial software (Prism 5 for Windows, Graph Pad Software). Data were investigated for normality with the D’Agostino & Pearson normality test. Data were presented as median and range. Duration of hospitalization, albumin and total protein concentrations were compared between groups by Mann–Whithney U test. Course of albumin and total protein concentration during hospitalization was evaluated by Friedman test and Dunn’s multiple comparison test. A value of *p* < 0.05 was considered statistically significant.

## 3. Results

### 3.1. Animals

Median age of dogs in group AA (6.0 years; 0.6–14.0 years) was not different from those of group CON (6.0 years; 0.6–15.0 years; *p* = 0.256). The median weight was significantly lower in group AA (11.5 kg; 1.0–56.7 kg) compared to the group CON (18.7 kg; 1.6–54.0 kg; *p* = 0.032).

Finally, medical records of 158 dogs were included in the analysis. These included 49 male intact, 21 male neutered, 50 female intact and 38 female neutered dogs. Median age of the dogs was 6 years (6 months–15 years). Median weight was 14.9 kg (1.0–56.7 kg). Most frequently represented breeds were mixed breed (53), Labrador Retriever (12), Pug (7), Chihuahua (6), Yorkshire Terrier (6), Golden Retriever (5), Bernese Mountain dog (4), Bichon Frisèe (4), German Sheepdog (4), Jack Russel Terrier (4), Dachshund (3), Maltese (3), Cavalier King Spaniel (3), the 44 remaining dogs belonged to various other breeds with less than 3 individuals per breed.

Causes of hypoalbuminemia in the dogs included intestinal loss of albumin (80), renal loss (34), loss through increased vascular permeability (15), reduced synthesis (9), reduced intake (4), and other causes (16) and were different between the study groups (*p* = 0.013; Table 1). The 158 dogs evaluated suffered from a wide variety of underlying diseases (Table 2).

**Table 1:** Comparison of demographic data and causes of hypoalbuminemia of 80 dogs with intravenous amino acid (AA) treatment and 78 dogs without amino acid treatment (CON).

	*n*	AA	*n*	CON	*p*
Age (years)	79	6.0 (0.6–14.0)	76	6.0 (0.6–15.0)	0.831
Weight (kg)	80	11.5 (1.0–56.7)	78	18.7 (1.6–54.0)	0.032*
Duration of hospitalization (days)	80	8 (3–33)	78	6 (3–24)	<0.001*
Tube feeding (*n*)	14		12		0.831
Causes of hypoalbuminemia:					0.013*
Intestinal loss	51		29		
Renal loss	9		25		
Increased vascular permeability Reduced synthesis	6		9		
Reduced synthesis	5		4		
Reduced intake	2		2		
Other	7		9		

AA, amino acid infused group; CON, group without amino acid treatment. *Significant differences; data are reported as median and range. Difference in age, weight duration of hospitalization and tube feeding was analyzed using the Mann–Whitney U test. Difference of cause of hypoalbuminemia was analyzed using Chi-square test.

**Table 2:** Underlying diseases of 158 hypoalbuminemic dogs.

Metabolic/endocrinologic	Neoplastic	Infectious/inflammatory	Immunologic	Other
Morbus Addison (2)	Lymphoma (4)	Acute Gastroenteritis (14)	PLN (24)	AHDS (41)
DKA (1)	Myeloma (1)	Sepsis (12)	PLE (17)	Intoxication (3)
PSS (1)	Sarcoma (1)	Pancreatitis (7)	IMHA (2)	Tetanus (3)
		Parvovirosis (5)		Post-surgical hemorrhage (1)
		Leptospirosis (4)		
		Babesiosis (2)		
		Hepatitis (2)		
		Polyarthritis (2)		
		Prostatitis (2)		
		Anaplasmosis (1)		
		Aspiration pneumonia (1)		
		Cholezystitis (1)		
		Coccidiosis (1)		
		Giardiose (1)		
		Leishmaniasis (1)		
		Wound infection (1)		

DKA, diabetic ketoacidosis; PSS, portosystemic shunt; IMHA, immunmediated hemolytic anemia; PLE, protein losing enteropathie; PLN, protein losing nephropathie; AHDS, acute hemorrhagic diarrhoe syndrome.

Dogs in group AA received a median dose of 9 g/100 kcal resting energy requirement (RER)/day (4–18 g/100 kcal RER/day) with a total amount of median 31 g/100 kcal RER (9–72 g/100 kcal RER) of a 10% amino acid solution intravenously over a period of 4 days (3–11 days). Tube feeding was performed in group AA (14/80 dogs) as well as in group CON (12/78 dogs; *p* = 0.831).

### 3.2. Survival

Of the 158 dogs, discharge was documented in 118 dogs. Forty dogs (33.9%) died or were euthanized during hospitalization. No difference in survival rate was found between group AA (62 dogs survived, 18 did not survive), and group CON (56 survived, 22 did not survive; *p* = 0.466). A difference in the median positive SIRS criteria at day 0 was neither observed between group AA (2; 0–5) and CON (2; 0–4; *p* = 0.645), nor between surviving dogs of group AA (3; 0–5) and group CON (2; 1–4; *p* = 0.282), or between non-surviving dogs of group AA (2; 1–4), and group CON (2.5; 0–4; *p* = 0.330).

Median duration of hospitalization in surviving and non-surviving dogs was longer in group AA (8 days; 3–33 days) compared to group CON (6 days; 3–24 days; *p* < 0.001). In surviving dogs, time of hospitalization was also longer in group AA (8 days; 4–33 days) compared to group CON (6.5 days; 3–24 days; *p* = 0.003). However, hospitalization time was not different between non-surviving dogs in group AA (8 days; 3–15 days) compared to non-surviving dogs in group CON (6 days; 3–11 days; *p* = 0.062).

### 3.3. Albumin and total protein concentration

Serum albumin concentration on day 0 was significantly lower in group AA compared to CON (*p* < 0.001). This difference stayed significant at all days with the exceptional day 2. On day 2, serum albumin concentration was not different between both groups (*p* = 0.134; Table 3).

**Table 3:** Albumin concentrations (median and range) in g/l in 80 dogs treated with amino acids (AA) and 78 dogs without amino acid treatment (CON) during hospitalization.

Day	AA		CON		*p*
	*n*	Albumin (g/l)	*n*	Albumin (g/l)	
0	80	18.1 (11.0–24.4)	78	21.6 (12.9–25.0)	<0.001*
1	26	17.9 (8.7–21.3)	21	21.3 (15.4–27.5)	<0.001*
2	55	19.0 (9.9–32.7)	44	20.3 (10.4–28.9)	0.134
3	33	18.5 (7.1–32.4)	28	22.4 (14.8–34.8)	<0.001*
4	18	18.5 (7.1–32.4)	13	21.6 (17.5–30.2)	0.037*
5	16	19.6 (13.3–27.2)	10	25.6 (23.1–31.3)	<0.001*

AA, amino acid infused group; CON, group without amino acid treatment. *Significant difference between median albumin levels in dogs treated with or without amino acids, analyzed using the Mann–Whitney U test.

Serum albumin concentrations in group AA were not different on each day of hospitalization compared to day 0 (*p* = 0.037). On *post hoc* Dunn’s multiple comparison test, no daily differences could be identified. In dogs of group CON, serum albumin concentrations were significantly higher on day 5 compared to day 0, to day 1 and to day 2 (*p* < 0.001).

Serum total protein concentration on day 0 was lower in group AA compared to CON (*p* < 0.001). This difference was also found on days 1 and 4. On day 2, 3, and 5, the serum total protein concentration was not different between AA and CON (Table 4). Serum total protein concentrations in group AA were higher on days 3 and 5 compared to day 1 (*p* = 0.002). In group CON serum total protein concentrations were different between the days of hospitalization (*p* = 0.031), but no difference between specific days could be found during *post hoc* analysis (Table 4).

**Table 4:** Total protein concentrations (median and range) in g/l in 80 dogs with amino acid infusion (AA) and 78 dogs without amino acid infusion (CON).

Day	AA		CON		*p*
	*n*	TP (g/l)	*n*	TP (g/l)	
0	58	34.4 (21.3–78.9)	68	43.3 (26.2–129.9)	<0.001*
1	14	29.7 (19.0–55.3)	12	40.5 (32.1–44.6)	0.006*
2	29	39.9 (24.0–66.6)	31	43.3 (25.6–66.6)	0.069
3	15	41.1 (28.7–68.8)	21	45.5 (36.0–116.1)	0.140
4	11	41.2 (28.6–53.1)	10	50.1 (37.2–81.3)	0.032*
5	9	44.5 (36.5–58.3)	7	47.7 (44.0–79.0)	0.211

AA, amino acid infused group; CON, group without amino acid treatment. *Significant difference between median albumin levels in dogs treated with or without amino acids analyzed using the Mann–Whitney U test.

Compared to day 0, serum albumin difference on day 2 was higher in group AA (0.9 g/L; −7.1–16.4 g/L) compared to group CON, where albumin decreased (−1.0 g/L; −8.9–13.7 g/L; *p* = 0.025). Serum albumin increase was higher in CON at day 5 compared to AA (*p* = 0.042). No difference in change of albumin concentration compared to day 0 was observed between groups at all other study points (Table 5).

**Table 5:** Difference in albumin concentration (median and range) in g/l in 80 dogs with amino acid infusion (AA) and 78 dogs without amino acid infusion (CON) during hospitalization.

Days	AA		CON		*p*
	*n*	Difference in albumin concentration (g/l)	*n*	Difference in albumin concentration (g/l)	
1 vs. 0	26	−0.7 (−3.3–5-5)	21	0.0 (−5.1–3.0)	0.700
2 vs. 0	55	0.9 (−7.1–16.4)	44	−1.0 (−8.9–13.7)	0.025*
3 vs. 0	33	0.7 (−11.5–10.2)	28	1.1 (−6.3–13.7)	0.783
4 vs. 0	18	1.5 (−5.0–7.7)	13	0.5 (−3.0–5.2)	0.496
5 vs. 0	16	1.6 (−4.5–12-3)	10	3.5 (2.0–9.3)	0.042*

AA, amino acid infused group; CON, group without amino acid treatment. *Significant differences in median difference in albumin concentration analyzed using the Mann–Whitney U test.

Albumin increased in 54 dogs in group AA and 52 dogs in group CON during hospitalization. On day 2 and day 5, significantly more dogs in group AA had an albumin increase of ≥ 1 g/L (26/55; 10/16) compared to group CON (11/44; 0/10; *p* = 0.036; *p* = 0.003). On day 2, significantly more dogs in group AA had an albumin increase of ≥ 5 g/L (8/55) compared to group CON (1/44; *p* = 0.041).

**Figure 2:**
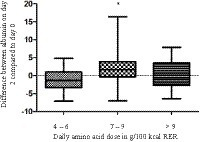
Albumin difference between day 2 and day 0 in 55 dogs after infusion of 10% amino acid solution at different median daily amino acid doses ranges. *Significant difference between 4–6 and 7–9 g amino acids/100 kcal RER.

Average daily amino acid dose of 6–9 g/100 kcal RER, but not <6 g/100 kcal RER, and not >9 g/100 kcal RER lead to an increase in albumin difference between day 0 and day 2 (*p* = 0.029; Figure 2). None of the cumulated amino acid dose ranges analyzed (<20 g/100 kcal RER; 20–30 g/100 kcal RER; 30–40 g/100 kcal RER; >40 g/100 kcal RER) was superior increasing albumin from day 0 to day 2 (*p* = 0.923).

### 3.4. Adverse effects of fluid therapy

Local adverse effects most likely associated with venous access or fluid therapy such as peri-phlebitis, pain or swelling at the catheter insertion side were observed in 15 dogs in group AA and 14 dogs in group CON (*p* = 0.836; Table 6).

**Table 6:** Complications at the catheter insertion side in 156 hypoalbuminemic dogs.

	AA	CON
Phlebitis	2	1
Paravenous infusion	10	12
Redness/swelling	3	1
Total	15	14

AA, amino acid infused group; CON, group without amino acid treatment.

## 4. Discussion

This retrospective study showed mild evidence of improvement in plasma albumin concentration in hypoalbuminemic dogs receiving 10% amino acid solution intravenously compared to dogs treated without amino acids. Serum albumin concentration, which was initially lower in AA, was not significantly different to CON at day 2. It should be noted that albumin values were not available for every patient on every day due to the retrospective character of the study. Albumin difference between day 0 and day 2 was more pronounced in AA compared to CON. The number of dogs with an albumin increase of ≥ 1 g/L and ≥ 5 g/L on day 2 was also higher in dogs in group AA. These findings suggest a positive effect of AA infusion.

However, lower initial albumin levels in the AA group limit the findings of the study. The standard treatment protocols in the clinic during the examination period recommended amino acid application at serum albumin concentration below 20 g/L which explains the different starting albumin levels.

In critically ill people, parenteral amino acid administration resulted in improved protein balance and protein synthesis (29, 30). In prematurely born children with low birth weight, the administration of high amounts of amino acids in combination with lipids resulted in a higher nitrogen balance and an increase in albumin and total body protein synthesis rate (34–37). However, despite potentially increased albumin synthesis, no increase in serum albumin concentration was observed.

Increased redistribution of albumin from the intravascular space to the extravascular space potentially blunted the positive effect of amino acid application in the dogs evaluated in the present study. This redistribution is especially pronounced in systemic inflammation and increased vascular permeability (1, 5, 40). Therefore, analysis of the albumin synthesis rate by measuring isotopes or nitrogen balance is sometimes recommended (41, 42). These analyses were not performed in the present study as it was a retrospective study. A study in humans calculated a duration of 8 days to increase the albumin concentration by 5 g/L (37). Only a few dogs in the present study received the 10% amino acid infusion for at least 8 days. However, albumin was increased by 5 g/L or more in 8/55 dogs in group AA on day 2.

An amino acid dose of 6–9 g/100 kcal RER per day led to an increase of albumin while lower or higher doses did not. Studies in very low birth weight infants receiving 2.4 mg/kg/d or 3.6 mg/kg/d amino acids found a higher albumin synthesis rate, protein synthesis rate and nitrogen balance in the group with a higher dose of amino acid administration (35, 36, 43). Protein balance, determined by nitrogen balance as well as by the leucine stable isotope method, was higher in infants receiving 3 g/kg/d amino acids instead of 1 g/kg/d (44). In adults with non-oliguric acute kidney injury (AKI), a higher amino acid intake (150 g/d versus 75 g/d) led to an increase in nitrogen balance (24). In the present study, only a low number of patients received the high amino acid dose >9 g/100 kcal RER. This can have contributed to the albumin increase in the middle dose group but not in the high dose group.

Veterinary studies evaluating the effect of amino acid infusion on albumin synthesis are rare. In rats, a reduced albumin transcription was observed during parenteral nutrition compared to enteral and oral nutrition (27). On the other hand, an increased albumin synthesis rate was detected after parenteral administration of amino acids in premature newborn pigs (33). Enteral nutrition is not always possible in critically ill animals, which is why parenteral nutrition is used in some cases. Albumin synthesis rate can be determined by analysis of albumin isotopes or nitrogen balance analyzed and this should be further evaluated in dogs in future studies (41, 42).

Mortality of hypoalbuminemic dogs in the present retrospective study was about 25%, and not different between groups. Malnutrition in critically ill patients causes severe physiological stress, leading to a change in hormone release and a resulting altered metabolic rate, altered use of energy sources, and decreased albumin production. Hypoalbuminemia is a negative prognostic parameter in critically ill dogs (45). In various scoring systems, such as Survival Prediction Index (SPI) I or II, or Acute Patient Physiologic and Laboratory Evaluation (APPLE) score, serum albumin concentration is included as a parameter to predict outcome (8, 46, 47).In the present study, only the SIRS criteria could be assessed retrospectively, which were not different between the groups.

In human patients, mortality was reduced by 50% by optimizing nutrition, defined by reaching individual protein and energy targets, while in critically ill humans, parenteral amino acid application, in addition to enteral nutrition, did not improve outcome (26, 48, 49). Outcome thus might not be influenced by amino acid application alone. A retrospective veterinary study evaluated the influence of the route of nutrition and the energy intake on hospital discharge. Discharge rate was higher in voluntary eating cats and dogs (92.9%) compared to the non-eating animals (38.4%). Animals receiving enteral or parenteral nutritional support had more severe diseases processes compared to voluntary eating animals. Analysis of cats and dogs with severe disease and intensive nutritional support showed a decrease in hospitalization time and higher discharge rate with increased energy intake (50). However, in the present study longer hospitalization time was observed in dogs receiving intravenous amino acid treatment. In human medicine a repeated calculation of the energy and protein intake, and an adapted nutritional therapy improved survival, which was accompanied with an increased hospitalization time (24). This could be caused by a potentially worse clinical condition and lower albumin concentrations of patients of the AA group compared to the CON group.

Local adverse effects of fluid therapy were also retrospectively assessed. Thrombophlebitis, redness, swelling, and paravenous infusion were observed in both groups, and no significant difference between the groups could be detected. Amino acids are hyperosmolar solutions with a theoretical osmolarity of 864 mosmoL/L. They can potentially cause tissue damage, especially after paravenous application. Osmolality is the crucial factor regarding to the tolerance of veins to fluids. Higher osmolality is associated with an increased risk of phlebitis. Humans infused with total parenteral nutrition (TPN) solutions without lipids with an osmolality of 920 mosmol/L had a 44% probability to develop phlebitis compared to humans receiving isotonic fluids (26%). The addition of a 10% lipid solution to the TPN decreased osmolarity (712 mosmol/L) and reduced the risk of phlebitis to 22% (51). Human case reports describe skin necrosis after extravasation of arginine monohydrochloride (52, 53). Necrosis at the jugular vein after extravasation of TPN is described in one cat (54). In a study of dogs comparing TPN with crystalloid therapy, 1/10 dogs in the TPN group developed phlebitis (55). The severity of the adverse effects was not recorded in the present study. The amino acid solution should ideally be administered via a central venous catheter to reduce the risks of paravenous infusion.

### 4.1. Limitations

The retrospective nature of the study and lack of uniform criteria for administration of amino acids are the major limitation of this study. The heterogenous study population and different initial albumin levels as day 0 between groups caused a bias in the comparability of the groups. Also, daily albumin concentrations were not available in all dogs. Due to the retrospective nature of the study, calculation of a severity score, such as the APPLE score to prove illness severity of the study groups was not possible.

## 5. Conclusion

This retrospective study showed mild evidence of improvement of albumin concentration in dogs with albumin ≤25 g/L treated with a 10% amino acid infusion compared to dogs not treated with amino acids. Intravenous amino acid application at doses of 6–9 g/100 kcal resting energy requirement/day over 3 days or longer might improve albumin synthesis. Further prospective studies with a standardized study and infusion protocol as well as standardized assessmet of the illness severty and cause of hypoalbuminemia are required to assess the effect of the amino acid infusion on albumin and total protein concentration.

## Data availability statement

The raw data supporting the conclusions of this article will be made available by the authors, without undue reservation.

## Ethics statement

Ethical review and approval was not required for the animal study because data were only retrospectively analyzed.

## Author contributions

SS, KH, and RD: conceptualization, investigation, and methodology. SS: data collection and writing original draft. SS and RD: formal analysis. KH and RD: supervision and writing review and editing. All authors contributed to the article and approved the submitted version.

## Conflict of interest

The authors declare that the research was conducted in the absence of any commercial or financial relationships that could be construed as a potential conflict of interest.

## Publisher’s note

All claims expressed in this article are solely those of the authors and do not necessarily represent those of their affiliated organizations, or those of the publisher, the editors and the reviewers. Any product that may be evaluated in this article, or claim that may be made by its manufacturer, is not guaranteed or endorsed by the publisher.
